# Supplementary material for: Pulmonary function and six-minute-walk test in patients after recovery from COVID-19: A prospective cohort study
Source: PLoS One. 2021 Sep 2;16(9):e0257040. doi: 10.1371/journal.pone.0257040 (PMC8412277; doi:10.1371/journal.pone.0257040)
Supplement: S1 Table — (DOCX) [file pone.0257040.s001.docx]

| Characteristics | Total  *(N=87)* | Mild symptoms  *(N=45)* | Non-severe pneumonia  *(N=35)* | Severe pneumonia  *(N=7)* | *P*-value |
| --- | --- | --- | --- | --- | --- |
| Laboratory results | | | | | |
| Oxygen saturation RA (%), mean ± SD | 98.1 ± 1.1 | 98.1 ± 1.1 | 98.0 ± 1.2 | 97.9 ± 1.1 | 0.846 |
| Hemoglobin (g/dl), mean ± SD | 13.4 ± 1.5 | 13.5 ± 1.8 | 13.4 ± 1.1 | 12.9 ± 0.6 | 0.616 |
| White blood cells (cells/mm^3^), mean ± SD | 6363 ± 1646 | 6428 ± 1730 | 6195 ± 1601 | 6782 ± 1397 | 0.646 |
| ALC (cells/mm^3^), mean ± SD | 2295 ± 745 | 2249 ± 723 | 2232 ± 686 | 2910 ± 991 | 0.073 |
| Platelet (/mm^3^), mean ± SD | 283068 ± 57489 | 285888 ± 61063 | 276228 ± 51781 | 299142 ±  64803 | 0.568 |
| LDH (U/l), mean ± SD | 161.6 ± 31.7 | 152.7± 23.7 | 168.9 ± 36.2 | 182.0 ± 38.9 | 0.014 |
| D-Dimer (ng/ml), median (IQR) | 245 (189-392) | 245 (189-328.5) | 220 (189-454) | 771 (190-831) | 0.003 |
| Creatinine (mg/dl), mean ± SD | 0.80 ± 0.18 | 0.81 ± 0.19 | 0.79 ± 0.17 | 0.75 ± 0.20 | 0.622 |
| CRP (mg/L), median (IQR) | 0.9 (0.5-2.3) | 0.7 (0.45-1.25) | 1.4 (0.4-2.3) | 2.6 (1.5-3.6) | 0.276 |

**S1 Table.** Laboratory results of 87 recovered COVID-19 patients during follow up period (60 day after onset of symptoms)

ALC: Absolute lymphocyte counts; RA: room air; LDH: Lactate dehydrogenase; CRP: C-reactive protein; SD: standard deviation; IQR: interquartile range.
