# Supplementary material for: Pulmonary function and six-minute-walk test in patients after recovery from COVID-19: A prospective cohort study
Source: PLoS One. 2021 Sep 2;16(9):e0257040. doi: 10.1371/journal.pone.0257040 (PMC8412277; doi:10.1371/journal.pone.0257040)
Supplement: S2 Table — (DOCX) [file pone.0257040.s002.docx]

**S2 Table**. Spirometry results of 87 recovered COVID-19 patients

| Patient  Number | Age (years) | BMI | Severity^*^ | FVC (% of predicted)^¶^ | | FEV1 (% of predicted)^¶^ | | FEV1/FVC (% ) | | FEF 25-75 (% of predicted)^¶^ | | PEF (% of predicted)^¶^ | |
| --- | --- | --- | --- | --- | --- | --- | --- | --- | --- | --- | --- | --- | --- |
|  |  |  |  | Pre^†^ | Post^†^ | Pre^†^ | Post^†^ | Pre^†^ | Post^†^ | Pre^†^ | Post^†^ | Pre^†^ | Post^†^ |
| 1  2  3  4  5  6  7  8  9  10  11  12  13  14  15  16  17  18  19  20  21  22  23  24  25  26  27  28  29  30  31  32  33  34  35  36  37  38  39  40  41  42  43  44  45  46  47  48  49  50  51  52  53  54  55  56  57  58  59  60  61  62  63  64  65  66  67  68  69  70  71  72  73  74  75  76  77  78  79  80  81  82  83  84  85  86  87 | 51  51  30  64  67  63  46  25  69  61  38  27  26  27  58  50  44  50  29  38  30  33  29  42  25  32  56  36  52  31  58  65  31  44  22  53  39  34  44  58  43  38  28  40  25  43  39  31  34  41  55  34  42  27  41  27  37  25  34  32  43  22  35  34  40  35  44  50  31  25  45  22  47  44  23  44  49  38  30  49  27  28  42  32  50  25  51 | 28.6  22.4  19.2  21.5  22.5  23.1  23.5  17.4  22.8  25.1  23.0  19.1  23.0  22.8  24.3  20.9  33.7  26.3  34.9  27.1  19.2  19.6  20.6  32.0  20.7  21.2  27.0  18.5  23.9  26.0  25.0  23.5  19.5  26.3  19.3  23.3  28.4  19.2  24.9  33.9  21.3  29.0  25.0  24.2  19.0  17.6  32.4  26.1  22.1  25.1  23.4  21.5  22.6  26.6  24.0  19.8  20.5  20.4  21.2  19.2  24.6  18.6  18.0  20.7  23.2  23.8  31.0  21.0  25.7  19.3  29.3  22.6  24.1  29.1  21.7  18.7  29.1  21.2  19.7  32.2  25.9  34.1  29.8  28.8  25.0  19.2  21.0 | 2  2  2  2  2  1  1  1  3  1  1  1  1  1  1  2  2  1  1  1  1  1  1  2  1  1  1  1  1  2  2  2  1  3  1  2  3  1  1  3  1  1  1  2  1  2  2  2  2  2  2  2  1  1  1  1  1  1  1  1  2  1  1  2  1  1  2  3  1  1  2  1  3  3  2  1  2  2  2  1  2  2  2  2  2  2  2 | 93  107  116  114  106  93  95  78  83  93  105  119  105  99  90  74  88  96  103  90  100  93  83  85  99  147  110  95  93  88  94  109  93  83  82  108  78  121  102  103  121  109  72  90  100  109  73  108  95  110  84  91  106  81  107  103  86  88  106  97  96  101  97  131  91  110  99  81  101  84  98  104  92  71  91  63  107  90  111  103  104  91  134  111  100  92  117 | 89  99  118  119  107  95  90  80  82  94  99  118  107  100  88  78  99  90  105  87  102  96  79  91  101  147  112  95  98  88  96  109  92  87  90  106  76  126  110  101  114  106  76  87  102  110  76  106  97  110  80  90  108  83  106  99  87  89  104  95  98  100  97  131  83  110  99  81  100  84  95  106  93  68  90  71  107  89  113  106  103  92  126  111  96  95  112 | 89  105  113  112  114  96  101  84  79  94  101  113  107  95  89  72  78  82  104  87  105  105  91  87  101  137  117  101  98  89  94  115  100  100  90  110  81  126  93  99  122  111  84  83  106  108  71  101  92  106  76  94  105  80  101  105  86  85  107  102  102  103  99  126  86  97  100  79  106  94  98  113  92  77  95  74  92  87  117  101  106  87  132  103  95  90  118 | 95  103  116  114  113  101  96  91  80  95  98  116  109  98  88  70  86  84  107  85  110  109  90  92  107  144  119  101  109  89  93  112  98  102  98  109  81  129  106  97  113  107  89  84  112  111  74  104  94  105  73  93  109  83  101  102  86  88  103  100  100  102  101  126  85  99  104  80  105  96  98  116  92  72  95  77  98  87  116  102  104  91  129  105  95  96  114 | 78  79  86  81  81  78  86  91  72  79  82  85  90  86  78  83  72  69  90  79  91  97  92  87  86  79  83  92  85  78  80  82  94  97  99  86  85  92  79  77  86  89  99  79  94  85  84  83  85  79  75  85  80  84  81  91  86  87  88  91  91  92  89  83  82  72  87  78  87  95  79  97  85  88  93  86  69  85  93  79  86  85  80  81  80  87  86 | 86  83  87  79  79  81  86  97  74  79  85  87  90  88  78  76  71  75  90  80  94  99  95  86  89  83  83  93  90  85  77  80  93  96  99  87  86  90  83  77  85  87  98  83  98  87  84  87  85  79  76  85  82  85  82  92  86  89  86  91  87  82  90  83  88  74  90  78  88  96  82  98  84  86  95  92  74  85  91  77  86  88  84  84  83  91  87 | 65  81  104  119  113  74  112  70  45  74  87  111  107  91  69  58  40  35  113  67  108  135  89  107  86  114  106  110  88  91  72  93  128  139  104  135  88  140  71  64  117  147  168  65  134  115  81  88  95  76  52  84  83  68  87  106  99  74  117  111  117  98  111  114  79  58  130  56  119  94  73  133  93  111  102  145  45  86  120  78  95  102  106  86  80  75  119 | 120  90  108  105  90  85  107  120  36  71  106  123  113  102  71  51  42  49  120  68  122  158  97  106  97  135  108  104  118  94  61  87  117  153  105  141  92  142  95  64  105  135  173  72  147  132  83  107  99  74  51  88  94  72  89  108  101  82  93  108  102  102  119  114  109  62  156  60  130  99  81  148  100  103  112  111  59  87  119  68  98  110  109  96  97  86  121 | 127  105  120  111  98  95  109  65  90  102  111  105  122  95  91  61  99  108  96  84  110  86  78  118  85  150  112  95  93  125  104  124  114  119  104  120  100  141  97  103  144  136  97  83  130  130  117  102  103  121  90  105  118  94  116  111  96  96  113  116  102  108  113  97  115  80  119  80  121  91  123  124  114  86  111  88  90  114  112  106  126  102  120  134  133  88  128 | 125  109  121  103  112  106  110  97  77  97  104  106  123  93  95  40  111  115  106  82  113  101  78  110  100  145  111  99  89  111  102  129  113  114  108  124  95  140  126  101  143  135  106  79  131  133  126  105  99  120  81  98  123  93  119  108  100  108  118  118  113  116  112  97  107  97  123  83  117  89  137  127  119  76  118  102  92  115  109  111  131  103  122  142  136  107  126 |

* Severity – 1= Mild symptom; 2 = Non-severe pneumonia; 3 = Severe pneumonia

^¶^ The results were expressed as a percentage of predicted values using normal values for the population of Thailand [Dejsomritrutai W, Nana A, Maranetra KN, Chuaychoo B, Maneechotesuwan K, Wongsurakiat P, et al. Reference spirometric values for healthy lifetime nonsmokers in Thailand. J Med Assoc Thai 2000; 83(5): 457-466.].

^†^ Prebronchodilator / Postbronchodilator

BMI: body mass index; FVC: forced vital capacity; FEV: forced expiratory volume in the first second; FEF: forced expiratory flow; PEF: peak expiratory flow.
